# The low-temperature structure of diethyl ether magnesium oxybromide

**DOI:** 10.1107/S1600536811043820

**Published:** 2011-10-29

**Authors:** Hannes Vitze, Hans-Wolfram Lerner, Michael Bolte

**Affiliations:** a Institut für Anorganische Chemie, J. W. Goethe-Universität Frankfurt, Max-von-Laue-Strasse 7, 60438 Frankfurt/Main, Germany

## Abstract

The crystal structure of the title compound, hexa-μ_2_-bromido-μ_4_-oxido-tetra­kis­[(diethyl ether)magnesium], [Mg_4_Br_6_O(C_4_H_10_O)_4_], determined from data measured at 173 K, differs from the previously known structure of diethyl ether magnesium oxybromide, which was determined from room-temperature data [Stucky & Rundle (1964 ▶). *J. Am. Chem. Soc*. **86**, 4821–4825]. The title compound crystallizes in the tetra­gonal space group *I* 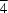, whereas the previously known structure crystallizes in a different tetra­gonal space group, namely *P* 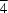
2_1_ *c*. Both molecules have crystallographic 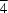 symmetry and show almost identical geometric parameters for the Mg, Br and O atoms. The crystal of the title compound turned out to be a merohedral twin emulating a structure with apparent Laue symmetry 4/*mmm*, whereas the correct Laue group is just 4/*m*. The fractional contribution of the minor twin component converged to 0.462 (1).

## Related literature

For Mg–Br complexes, see: Lerner (2005 ▶); Lerner *et al.* (2003 ▶); Metzler *et al.* (1994 ▶). For a polymorph of the title compound, see: Stucky & Rundle (1964 ▶). For the Cambridge Structural Database, see: Allen (2002 ▶).
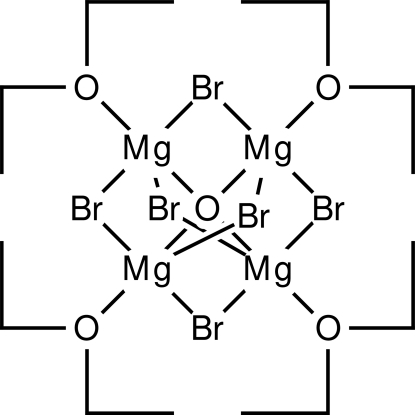


## Experimental

### 

#### Crystal data

[Mg_4_Br_6_O(C_4_H_10_O)_4_]
*M* *_r_* = 889.18
Tetragonal, 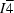

*a* = 10.4630 (13) Å
*c* = 15.276 (2) Å
*V* = 1672.3 (4) Å^3^
*Z* = 2
Mo *K*α radiation
μ = 7.30 mm^−1^
*T* = 173 K
0.25 × 0.22 × 0.18 mm

#### Data collection

Stoe IPDS II two-circle diffractometer
Absorption correction: multi-scan [*MULABS* (Spek, 2009 ▶; Blessing, 1995 ▶)] *T*_min_ = 0.169, *T*_max_ = 0.269
3746 measured reflections
1479 independent reflections
1455 reflections with *I* > 2σ(*I*)
*R*_int_ = 0.044

#### Refinement

*R*[*F* ^2^ > 2σ(*F* ^2^)] = 0.026
*wR*(*F* ^2^) = 0.067
*S* = 1.08
1479 reflections
73 parameters
H-atom parameters constrained
Δρ_max_ = 0.62 e Å^−3^
Δρ_min_ = −0.56 e Å^−3^
Absolute structure: Flack (1983 ▶), 689 Friedel pairs
Flack parameter: −0.02 (2)

### 

Data collection: *X-AREA* (Stoe & Cie, 2001 ▶); cell refinement: *X-AREA*; data reduction: *X-AREA*; program(s) used to solve structure: *SHELXS97* (Sheldrick, 2008 ▶); program(s) used to refine structure: *SHELXL97* (Sheldrick, 2008 ▶); molecular graphics: *XP* (Sheldrick, 2008 ▶); software used to prepare material for publication: *SHELXL97*.

## Supplementary Material

Crystal structure: contains datablock(s) I, global. DOI: 10.1107/S1600536811043820/bh2385sup1.cif

Structure factors: contains datablock(s) I. DOI: 10.1107/S1600536811043820/bh2385Isup2.hkl

Additional supplementary materials:  crystallographic information; 3D view; checkCIF report

## Figures and Tables

**Fig. 1.**
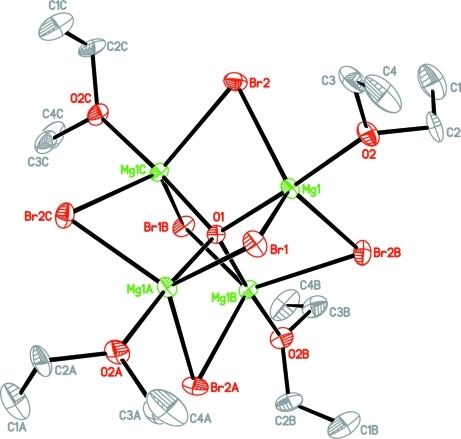
Perspective view of the title compound with displacement ellipsoids at the 50% probability level. H atoms are omitted for clarity. Symmetry operators for generating equivalent atoms: (A): 1 - x, -y z; (B): 1/2 + y, 1/2 - x, 1/2 - z; (C): 1/2 - y, -1/2 + x, 1/2 - z.

## supplementary crystallographic information

### Comment

The solid-state structures of Mg–Br compounds feature coordination numbers of the Mg center from four as in [MgBr(Si*^t^*Bu_3_)(THF)]_2_ (Lerner *et al.*, 2003; Lerner, 2005) to six as in [MgBr_2_(THF)_4_] (Metzler *et al.*, 1994). Most of the Mg–Br compounds possess an octahedral coordination sphere which surrounds the Mg cation whereas only a few compounds are found in the Cambridge Structural Database (Allen, 2002) with five-coordinated Mg centers as found in the solid-state structure of the title compound. We report here the X-ray crystal structure analysis of [(MgOEt_2_)_4_Br_6_O], which could be isolated from a solution of C_6_F_5_MgBr in Et_2_O.

Data for the crystal structure of the title compound were collected at 173 K. It crystallizes in the tetragonal space group *I*4 with crystallographic 4 symmetry. The previously known polymorph (Stucky & Rundle, 1964) for which data were collected at room temperature crystallizes in the space group *P*42_1_*c* and has crystallographic 4 symmetry, too. However, in the latter structure there is severe disorder of the C atoms, whereas in the title compound, no disorder was found. The geometric parameters involving Mg, Br and O atoms agree well in both structures.

Since the structures show striking similarities and were measured at different temperatures, a phase transition between them cannot be excluded.

### Experimental

To a suspension of Mg turnings (0.5 g, 20.2 mmol) in 25 ml Et_2_O, 2.3 ml C_6_F_5_Br is added dropwise. The reaction starts when 0.3 ml of C_6_F_5_Br have been added. The rest of C_6_F_5_Br is added dropwise at such a rate that the reaction mixture remains at its boiling point and refluxing is continued for 1 h until the magnesium turnings have dissolved completely. During the storing of this solution for 3 weeks, colorless crystals of the title compound were grown at room temperature.

### Refinement

H atoms could be located in a difference Fourier map, but they were refined using a riding model with isotropic displacement parameters *U*_iso_(H) set to 1.2*U*_eq_(C_methylene_) and C—H = 0.99 Å or *U*_iso_(H) set to 1.5*U*_eq_(C_methyl_) and C—H = 0.98 Å. The crystal turned out to be a merohedral twin emulating a structure with Laue symmetry 4/*mmm*. The twin law (0 1 0/1 0 0/0 0 1) is a twofold rotation about the diagonal between the *a* and *b* axis and the fractional contribution of the minor twin component refined to 0.462 (1).

### Crystal data

**Table:**

[Mg_4_Br_6_O(C_4_H_10_O)_4_]	*D*_x_ = 1.766 Mg m^−^^3^
*M**_r_* = 889.18	Mo *K*α radiation, λ = 0.71073 Å
Tetragonal, *I*4	Cell parameters from 3746 reflections
Hall symbol: I -4	θ = 3.8–25.6°
*a* = 10.4630 (13) Å	µ = 7.30 mm^−^^1^
*c* = 15.276 (2) Å	*T* = 173 K
*V* = 1672.3 (4) Å^3^	Block, colourless
*Z* = 2	0.25 × 0.22 × 0.18 mm
*F*(000) = 868	

### Data collection

**Table:**

Stoe IPDS II two-circle diffractometer	1479 independent reflections
Radiation source: fine-focus sealed tube	1455 reflections with *I* > 2σ(*I*)
graphite	*R*_int_ = 0.044
ω scans	θ_max_ = 25.3°, θ_min_ = 3.8°
Absorption correction: multi-scan (*MULABS*; Spek, 2009; Blessing, 1995)	*h* = −12→12
*T*_min_ = 0.169, *T*_max_ = 0.269	*k* = −8→12
3746 measured reflections	*l* = −13→18

### Refinement

**Table:**

Refinement on *F*^2^	Hydrogen site location: inferred from neighbouring sites
Least-squares matrix: full	H-atom parameters constrained
*R*[*F*^2^ > 2σ(*F*^2^)] = 0.026	*w* = 1/[σ^2^(*F*_o_^2^) + (0.0427*P*)^2^ + 0.284*P*] where *P* = (*F*_o_^2^ + 2*F*_c_^2^)/3
*wR*(*F*^2^) = 0.067	(Δ/σ)_max_ < 0.001
*S* = 1.08	Δρ_max_ = 0.62 e Å^−^^3^
1479 reflections	Δρ_min_ = −0.56 e Å^−^^3^
73 parameters	Extinction correction: *SHELXL97* (Sheldrick, 2008), Fc^*^=kFc[1+0.001xFc^2^λ^3^/sin(2θ)]^-1/4^
0 restraints	Extinction coefficient: 0.0036 (4)
0 constraints	Absolute structure: Flack (1983), with 689 Friedel pairs
Primary atom site location: structure-invariant direct methods	Flack parameter: −0.02 (2)
Secondary atom site location: difference Fourier map	

### Fractional atomic coordinates and isotropic or equivalent isotropic displacement parameters (Å^2^)

**Table:**

	*x*	*y*	*z*	*U*_iso_*/*U*_eq_	
Mg1	0.40713 (19)	0.12350 (19)	0.32357 (13)	0.0135 (5)	
Br1	0.5000	0.0000	0.45892 (4)	0.01815 (19)	
Br2	0.19607 (5)	0.04477 (5)	0.24533 (6)	0.02114 (18)	
O1	0.5000	0.0000	0.2500	0.0120 (13)	
C1	0.2012 (10)	0.4180 (8)	0.3236 (7)	0.044 (2)	
H1A	0.1953	0.5113	0.3196	0.065*	
H1B	0.1161	0.3823	0.3348	0.065*	
H1C	0.2345	0.3836	0.2685	0.065*	
C2	0.2906 (8)	0.3818 (7)	0.3982 (6)	0.0286 (17)	
H2A	0.3759	0.4195	0.3875	0.034*	
H2B	0.2573	0.4174	0.4538	0.034*	
O2	0.3026 (5)	0.2433 (5)	0.4064 (3)	0.0210 (10)	
C3	0.2032 (8)	0.1864 (7)	0.4613 (5)	0.0289 (16)	
H3A	0.1932	0.0953	0.4453	0.035*	
H3B	0.1210	0.2299	0.4495	0.035*	
C4	0.2326 (9)	0.1958 (10)	0.5579 (5)	0.040 (2)	
H4A	0.1629	0.1570	0.5915	0.061*	
H4B	0.2413	0.2859	0.5744	0.061*	
H4C	0.3126	0.1507	0.5704	0.061*	

### Atomic displacement parameters (Å^2^)

**Table:**

	*U*^11^	*U*^22^	*U*^33^	*U*^12^	*U*^13^	*U*^23^
Mg1	0.0173 (10)	0.0158 (10)	0.0075 (9)	0.0031 (8)	0.0010 (8)	−0.0015 (8)
Br1	0.0265 (7)	0.0226 (6)	0.0054 (3)	0.0057 (6)	0.000	0.000
Br2	0.0149 (3)	0.0285 (3)	0.0200 (3)	0.0026 (2)	−0.0018 (4)	−0.0064 (4)
O1	0.0150 (18)	0.0150 (18)	0.006 (3)	0.000	0.000	0.000
C1	0.056 (5)	0.035 (4)	0.040 (5)	0.020 (4)	−0.001 (5)	0.002 (4)
C2	0.038 (4)	0.015 (3)	0.032 (4)	0.010 (3)	0.004 (3)	−0.008 (3)
O2	0.024 (2)	0.023 (2)	0.015 (2)	0.005 (2)	0.008 (2)	−0.0037 (19)
C3	0.029 (4)	0.027 (4)	0.031 (4)	0.009 (3)	0.013 (3)	0.001 (4)
C4	0.051 (6)	0.056 (6)	0.014 (4)	0.027 (5)	0.009 (3)	−0.004 (4)

### Geometric parameters (Å, °)

**Table:**

Mg1—O1	1.969 (2)	C1—H1A	0.9800
Mg1—O2	2.090 (5)	C1—H1B	0.9800
Mg1—Br2^i^	2.597 (2)	C1—H1C	0.9800
Mg1—Br1	2.625 (2)	C2—O2	1.460 (8)
Mg1—Br2	2.643 (2)	C2—H2A	0.9900
Mg1—Mg1^i^	3.206 (3)	C2—H2B	0.9900
Mg1—Mg1^ii^	3.206 (3)	O2—C3	1.463 (9)
Mg1—Mg1^iii^	3.233 (4)	C3—C4	1.510 (11)
Br1—Mg1^iii^	2.625 (2)	C3—H3A	0.9900
Br2—Mg1^ii^	2.597 (2)	C3—H3B	0.9900
O1—Mg1^iii^	1.9689 (19)	C4—H4A	0.9800
O1—Mg1^i^	1.969 (2)	C4—H4B	0.9800
O1—Mg1^ii^	1.969 (2)	C4—H4C	0.9800
C1—C2	1.523 (12)		
			
O1—Mg1—O2	175.84 (18)	Mg1—O1—Mg1^i^	109.01 (6)
O1—Mg1—Br2^i^	88.40 (7)	Mg1^iii^—O1—Mg1^ii^	109.01 (6)
O2—Mg1—Br2^i^	95.71 (15)	Mg1—O1—Mg1^ii^	109.01 (6)
O1—Mg1—Br1	86.78 (7)	Mg1^i^—O1—Mg1^ii^	110.39 (12)
O2—Mg1—Br1	90.70 (16)	C2—C1—H1A	109.5
Br2^i^—Mg1—Br1	118.15 (8)	C2—C1—H1B	109.5
O1—Mg1—Br2	87.12 (7)	H1A—C1—H1B	109.5
O2—Mg1—Br2	91.33 (16)	C2—C1—H1C	109.5
Br2^i^—Mg1—Br2	120.44 (8)	H1A—C1—H1C	109.5
Br1—Mg1—Br2	120.81 (8)	H1B—C1—H1C	109.5
O1—Mg1—Mg1^i^	35.49 (3)	O2—C2—C1	111.3 (6)
O2—Mg1—Mg1^i^	148.63 (17)	O2—C2—H2A	109.4
Br2^i^—Mg1—Mg1^i^	52.92 (7)	C1—C2—H2A	109.4
Br1—Mg1—Mg1^i^	103.98 (7)	O2—C2—H2B	109.4
Br2—Mg1—Mg1^i^	103.99 (9)	C1—C2—H2B	109.4
O1—Mg1—Mg1^ii^	35.49 (3)	H2A—C2—H2B	108.0
O2—Mg1—Mg1^ii^	142.70 (17)	C2—O2—C3	113.0 (6)
Br2^i^—Mg1—Mg1^ii^	106.54 (9)	C2—O2—Mg1	126.1 (5)
Br1—Mg1—Mg1^ii^	103.98 (7)	C3—O2—Mg1	118.4 (4)
Br2—Mg1—Mg1^ii^	51.63 (7)	O2—C3—C4	112.9 (7)
Mg1^i^—Mg1—Mg1^ii^	60.57 (7)	O2—C3—H3A	109.0
O1—Mg1—Mg1^iii^	34.80 (6)	C4—C3—H3A	109.0
O2—Mg1—Mg1^iii^	142.56 (16)	O2—C3—H3B	109.0
Br2^i^—Mg1—Mg1^iii^	104.34 (9)	C4—C3—H3B	109.0
Br1—Mg1—Mg1^iii^	51.98 (4)	H3A—C3—H3B	107.8
Br2—Mg1—Mg1^iii^	104.66 (9)	C3—C4—H4A	109.5
Mg1^i^—Mg1—Mg1^iii^	59.72 (4)	C3—C4—H4B	109.5
Mg1^ii^—Mg1—Mg1^iii^	59.72 (4)	H4A—C4—H4B	109.5
Mg1^iii^—Br1—Mg1	76.05 (9)	C3—C4—H4C	109.5
Mg1^ii^—Br2—Mg1	75.45 (9)	H4A—C4—H4C	109.5
Mg1^iii^—O1—Mg1	110.39 (12)	H4B—C4—H4C	109.5
Mg1^iii^—O1—Mg1^i^	109.01 (6)		
			
O1—Mg1—Br1—Mg1^iii^	0.0	Br2^i^—Mg1—O1—Mg1^ii^	−121.97 (10)
O2—Mg1—Br1—Mg1^iii^	176.68 (18)	Br1—Mg1—O1—Mg1^ii^	119.72 (9)
Br2^i^—Mg1—Br1—Mg1^iii^	−86.46 (9)	Br2—Mg1—O1—Mg1^ii^	−1.38 (7)
Br2—Mg1—Br1—Mg1^iii^	84.71 (9)	Mg1^i^—Mg1—O1—Mg1^ii^	−120.57 (8)
Mg1^i^—Mg1—Br1—Mg1^iii^	−31.31 (7)	Mg1^iii^—Mg1—O1—Mg1^ii^	119.72 (9)
Mg1^ii^—Mg1—Br1—Mg1^iii^	31.31 (7)	C1—C2—O2—C3	−86.8 (8)
O1—Mg1—Br2—Mg1^ii^	1.02 (5)	C1—C2—O2—Mg1	74.9 (8)
O2—Mg1—Br2—Mg1^ii^	−175.11 (18)	Br2^i^—Mg1—O2—C2	17.5 (6)
Br2^i^—Mg1—Br2—Mg1^ii^	87.47 (9)	Br1—Mg1—O2—C2	135.9 (6)
Br1—Mg1—Br2—Mg1^ii^	−83.50 (9)	Br2—Mg1—O2—C2	−103.3 (6)
Mg1^i^—Mg1—Br2—Mg1^ii^	32.52 (7)	Mg1^i^—Mg1—O2—C2	16.9 (8)
Mg1^iii^—Mg1—Br2—Mg1^ii^	−29.32 (7)	Mg1^ii^—Mg1—O2—C2	−109.6 (6)
Br2^i^—Mg1—O1—Mg1^iii^	118.31 (8)	Mg1^iii^—Mg1—O2—C2	140.2 (5)
Br1—Mg1—O1—Mg1^iii^	0.0	Br2^i^—Mg1—O2—C3	178.3 (5)
Br2—Mg1—O1—Mg1^iii^	−121.10 (8)	Br1—Mg1—O2—C3	−63.3 (5)
Mg1^i^—Mg1—O1—Mg1^iii^	119.72 (9)	Br2—Mg1—O2—C3	57.5 (5)
Mg1^ii^—Mg1—O1—Mg1^iii^	−119.72 (9)	Mg1^i^—Mg1—O2—C3	177.7 (4)
Br2^i^—Mg1—O1—Mg1^i^	−1.40 (7)	Mg1^ii^—Mg1—O2—C3	51.2 (6)
Br1—Mg1—O1—Mg1^i^	−119.72 (4)	Mg1^iii^—Mg1—O2—C3	−59.0 (6)
Br2—Mg1—O1—Mg1^i^	119.19 (10)	C2—O2—C3—C4	−82.1 (8)
Mg1^ii^—Mg1—O1—Mg1^i^	120.57 (8)	Mg1—O2—C3—C4	114.7 (6)
Mg1^iii^—Mg1—O1—Mg1^i^	−119.72 (9)		

Symmetry codes: (i) *y*+1/2, −*x*+1/2, −*z*+1/2; (ii) −*y*+1/2, *x*−1/2, −*z*+1/2; (iii) −*x*+1, −*y*, *z*.
